# A no-go related prefrontal negativity larger to irrelevant stimuli that are difficult to suppress

**DOI:** 10.1186/1744-9081-5-25

**Published:** 2009-06-25

**Authors:** Alice M Proverbio, Marzia Del Zotto, Nicola Crotti, Alberto Zani

**Affiliations:** 1 Dept. of Psychology, University of Milano-Bicocca, Milan, Italy; 2 San Raffaele Hospital, HSR Monte Tabor Foundation, Milan, Italy; 3 Inst. of Bioimaging and Molecular Physiology, CNR, Milano-Segrate, Italy

## Abstract

**Background:**

There is a wide debate in the literature about whether N2/P3 effects in no-go trials reflect the inhibition of an intended action, or the absence of a negative movement-related potential typical of go trials. The aim of this study was to provide an objective measure of the suppression of irrelevant information (in a conjoined selective visual attention task) under conditions that were perfectly comparable from the viewpoint of the motoric processes involved.

**Methods:**

Twenty-nine right-handed students took part in the study. Their EEGs were recorded from 128 scalp sites while they viewed gratings of four different spatial frequencies (from 0.75 to 6 c/deg) randomly flashed in the four upper and lower quadrants of the visual field. The tasks consisted of attending and responding to a conjunction of spatial frequency and space location. Intermediate frequencies (1.5 and 3 c/deg) acted as distracters or lures. Analysis of the ERPs elicited by the same physical stimulus, close in spatial frequency to the actual target and falling within the attended quadrant (pseudo-target) vs. a non-target location, allowed us to identify the time course and neural bases of brain activation during the suppression of irrelevant information.

**Results:**

FAs were on average 9% for pseudo-targets and 0.2% for other types of lures, indicating that the former were more difficult to suppress. Target-related ERP components (occipito/temporal *selection negativity*, posterior P3b and precentral motor N2) were greater to pseudo-targets than other distracters. A large prefrontal negativity (370–430 ms) was also identified, much larger to pseudo-targets than non-targets (and absent in response to real targets), thus reflecting response inhibition and top-down cognitive control processes.

**Conclusion:**

A LORETA inverse solution identified the neural generators of this effect in the left dorsolateral prefrontal cortex (DLPF), left and right fusiform gyri and bilateral superior temporal cortices. The tentative hypothesis is advanced that these activations might reflect the modulatory effects exerted by the fronto/temporal circuit for the suppression of irrelevant information.

## Background

One of the main problems in investigating cognitive or motor suppression processes in go/no-go tasks is that, while go trials are associated with response motor preparation and execution, no-go trials are not, so it is difficult to establish which components are related to response/stimulus suppression typical of no-go trials and which depend on the motor and decision-making processes typical of go trials. Some authors have tried to overcome this problem by comparing motor with count conditions [1-4] or with saccadic eye movements [5] in go trials, or by varying the degree of effort required to withhold the go response [6], or by using a hybrid choice-reaction go/no-go procedure involving selective response priming [7]. The overall pattern of results consists of a fronto-central negative wave (N2) peaking at about 200–400 ms and an increased frontal P3 response to no-go trials [2,8,9] thought to represent inhibition of responses with no-go stimuli. N2 is sometimes interpreted as a clear sign of response inhibition with a generator in the anterior cingulate cortex [8], and at other times as a non-motoric stage of inhibition, or recognition of the need for inhibition [1]. In other studies, the fronto/central P3 to no-go trials has been associated with response inhibition, generated in the anterior cingulate cortex [10]. Furthermore, the functional significance of N2/P3 effects is debated, since they may represent purely motor inhibition, detection of response conflict, differences in attentional allocation or cognitive inhibition processes.

Notwithstanding the wide literature on this matter, there seems to be no convergence of interpretation. Indeed, Verleger and coauthors [11], who addressed the question whether no-go P3 reflected inhibition of the intended action or resulted from the absence of a negative movement-related potential typical of go trials, advanced the hypothesis that the no-go P3 might reflect monitoring of the withdrawal from overt action, and could be interpreted as the inverse of the negative motor potentials characteristic of go trials. Again, Salisbury and coauthors [4] tried to disentangle the effects of button press on the amplitude of the P3 component using an auditory go/no-go task in which P300 was measured on button-press and silent-count tasks in control subjects. An estimate of motor activity was constructed from a simple reaction time task, and the motor estimate was subtracted from the button-press P300 according to Kok's formula [9]: true P300 = go P300-motor potentials. The results showed that P300 was smaller and its topography different in the button-pressing task compared to silent-counting, while the motor-correction procedure generated a P300 with normal topography. The authors concluded that no-go P300 responses in button-pressing tasks are confounded by motor potentials, and that motor potential contamination is a real and insidious confounder, which must be dealt with when addressing response inhibition tasks. Overall, the whole issue remains far from understood or resolved because of the intrinsic differences between go and no-go conditions in the oddball paradigm.

The aim of this study was to provide an objective measure of the suppression of non-target stimuli in a conjoined selective visual attention task involving processing of different types of distracters more or less similar to targets, and therefore more or less difficult to suppress. By comparing brain activity relative to pseudo-targets (to which subjects did not actually respond) with that relative to non-targets falling at a non-attended location (easier to suppress), we identified the neural bases of the mechanism by which irrelevant information is suppressed under conditions perfectly comparable from the viewpoint of the motoric processes involved. Gratings of four different spatial frequencies (from 0.75 to 6 c/deg) were repeatedly flashed in the four upper and lower quadrants of the left and right visual fields and intermediate frequencies acted as distracters (never being targets in spatial frequency). Analysis of the ERP components elicited by the same physical stimulus, close in spatial frequency to the actual target and falling within the attended vs. unattended location, allowed us to identify the time course and neural bases of brain activation during the suppression of irrelevant information.

## Methods

### Participants

Twenty-nine university students (13 males and 16 females) ranging in age from 20 to 30 years (mean age = 23 years) took part in this experiment as volunteers. All participants had a normal or corrected-to-normal vision with right eye dominance. They were strictly right-handed as assessed by the Edinburgh Inventory and none of them had any left-handed relatives. The experiments were conducted with the understanding and written consent of each participant according to the Declaration of Helsinki (BMJ 1991; 302: 1194) with approval from the Ethical Committee of the Italian National Research Council (CNR) and in compliance with APA ethical standards for the treatment of human volunteers (1992, American Psychological Association). Subjects gained academic credits for their participation. Three subjects were subsequently discarded because of excessive eye-movements.

### Stimuli and procedure

Participants were seated in a dimly lit, electrically shielded cubicle and gazed binocularly on a fixation point permanently present in the centre of a visual display situated 114 cm in front of them. They were instructed to avoid any kind of eye or body movement. Four square-wave luminance-modulated vertical gratings of 0.75, 1.5, 3, 6 c/deg were randomly presented for 80 ms in the four quadrants of the visual field. The rectangular patterns were replaced for an interval varying randomly between 690 and 790 ms (SOA 770–870 ms) with an isoluminant grey field (35 cd/m^2^). Stimulus and background had equal average luminance to avoid flash stimulation. The mean grating luminance was measured for each spatial frequency and space location. An ANOVA performed on the luminance values showed no significant difference, thus proving stimulus equiluminance (43 cd/m^2^).

The gratings were randomly presented in pattern-onset mode within the left and right upper and lower hemifields of a PC screen. Within each hemifield, the grating stimulation began 2.5° above or below the horizontal meridian, and 1.5° lateral to the vertical meridian, and extended to 3.5° above or below the horizontal meridian and 5° along it. Different conjoined selective attention conditions were administered in random order for 0.75 or 6 c/deg within each hemifield to each subject. Irrespective of target frequency, gratings of 1.5 and 3 c/deg always served as potential distracters. Before the beginning of each task condition, the participants were instructed to pay conjoined attention to a spatial frequency within a given hemifield (e.g. 6 c/deg in the right upper field) and to ignore the other combinations of frequencies and hemifields. Thus, although the physical stimuli remained unchanged, attention shifted across spatial frequency and space location. While intermediate frequencies falling in an attended quadrant could share the space relevance with actual targets, their frequency relevance depended on their similarity with the latter. In detail, the 3 c/deg gratings, being 1 octave below 6 c/deg (an octave change in spatial frequency doubles or halves the frequency), fell close to the 6/deg spatial frequency bandwidth sensitivity, while 1.5 fell close or within the 0.75 spatial frequency bandwidth sensitivity, as demonstrated by psychophysical and VEP studies [12,13]. Evidence for a bandwidth of approximately an octave was originally provided by adaptation and masking studies [14,15]. Blakemore and Campbell [14] found that after prolonged observation of a high-contrast sinusoidal grating, gratings of similar spatial frequency were harder to detect; more contrast was needed to see them at threshold. This threshold elevation effect was strongest for test gratings that matched the adapting frequency, and the effect fell to half strength at about 0·5 octave either side of the adapting frequency – hence, a bandwidth of 1 octave. Weak effects were observed with test gratings approaching about 2 octaves below and 1·25 octaves above the adaptation frequency.

For this reason we assumed that frequency relevance might also affect intermediate stimuli, as shown in previous electrophysiological studies [12].

Thus, the same stimulus under different attention conjunction conditions could be: (i) relevant in both spatial location and spatial frequency (pseudo-target), when it fell in the target quadrant and its spatial frequency was 1.5 for 0.75 c/deg targets or 3 for 6/deg targets; (ii) relevant in spatial location but irrelevant in spatial frequency (L+F-), for 1.5 c/deg gratings when 6 c/deg was the target and for 3 c/deg when 0.75 c/deg was the target; (iii) irrelevant in spatial location but relevant in spatial frequency (L-F+/-); or (iv) irrelevant in both features (L-F-), according to the paradigm devised by Zani and Proverbio [16].

To monitor spatial and stimulus attention selectivity, the volunteers were instructed to press a button in response to targets as accurately and quickly as possible, allowing their reaction times (RT) to be recorded as well. In half the blocks, the participants pushed the detection-RT button with the index finger of the left hand, whereas in the other half they used the right hand. The order of hands was counterbalanced across participants. The order with which the attention tasks were administered and spatial locations attended was counterbalanced across participants and experimental sessions.

### EEG recording and analysis

The EEG was continuously recorded from 128 scalp sites according to the extended international 10–5 system [17] using an elastic cap embedded with tin electrodes. The sampling rate was 512 Hz. Vertical eye movements were recorded by two electrodes placed below and above the right eye, while horizontal movements were recorded from electrodes placed at the outer canthi of the eyes. Linked ears served as the reference lead. The EEG and electro-oculogram (EOG) were amplified with a half-amplitude band pass of 0.016–100 Hz. Electrode impedance was kept below 5 kΩ. EEG epochs were synchronized with the onset of stimulus presentation and analyzed by ANT-EEProbe software. Computerized artefact rejection was performed before averaging to discard epochs in which eye movements, blinks, excessive muscle potentials or amplifier blocking occurred. EEG epochs associated with an incorrect behavioural response were also excluded. The artefact rejection criterion was a peak-to-peak amplitude exceeding 50 μV, and the rejection rate was ~5%. ERPs were averaged offline from -200 ms before to 800 ms after stimulus onset. ERP components were identified and measured with reference to the average baseline voltage over the interval -100 ms to 0 ms relative to stimulus onset.

Low Resolution Electromagnetic Tomography (LORETA [18]) was performed on ERP difference waves at various time latencies. LORETA, which is a discrete linear solution to the inverse EEG problem, corresponds to the 3D distribution of electric neuronal activity that has maximum similarity (i.e. maximum synchronization), in terms of orientation and strength, between neighbouring neuronal populations (represented by adjacent voxels). In this study an improved version of standardized low-resolution brain electromagnetic tomography (sLORETA) was used, which incorporates a singular value decomposition-based lead field weighting: swLORETA [19]. Source space properties were: grid spacing = 10 mm; estimated SNR = 3.

Distinct ERP averages were obtained for each electrode site, grating spatial frequency, space location, and conjoined-attention condition. Grand-average ERPs were further computed independently of physical stimulus parameters (retinal coordinates and spatial frequency). In this study, only ERPs to intermediate non-target frequencies (i.e. 1.5 and 3 c/deg) were analyzed under the various attention conditions, to show the effect of neural suppression of irrelevant stimuli bearing different degrees of similarity to targets. Comparisons were also made with ERPs to effective targets, but an in-depth discussion of attention effects for 0.75 and 6 c/deg gratings can be found elsewhere [20].

ERP components were quantified by automatically measuring their mean amplitudes across time within the following latency ranges: 230–270 ms for the occipito-temporal N2 (*selection negativity*) at P9 and P10 sites, 275–315 ms for the N2 motor potential at FCC1h and FCC2h sites, 370–430 ms for the prefrontal NP400 component at PF1 and PF2 sites, and 380–500 ms for the posterior P3b component at PPO1 and PPO2 sites.

Separate two-way repeated-measure analyses of variance (ANOVAs) were performed on the mean values computed for each individual subject as a function of the attention condition, and independent of physical stimulus parameters. Factors were: attentional relevance (L+ F+/-, L+F-, L-F+/-, L-F-) and cerebral hemisphere (right and left). Possible type 1 errors associated with inhomogeneity of variance were controlled by the Greenhouse-Geisser procedure. Post-hoc Tukey tests were used for multiple comparisons of means.

## Results

**Figure 1:**
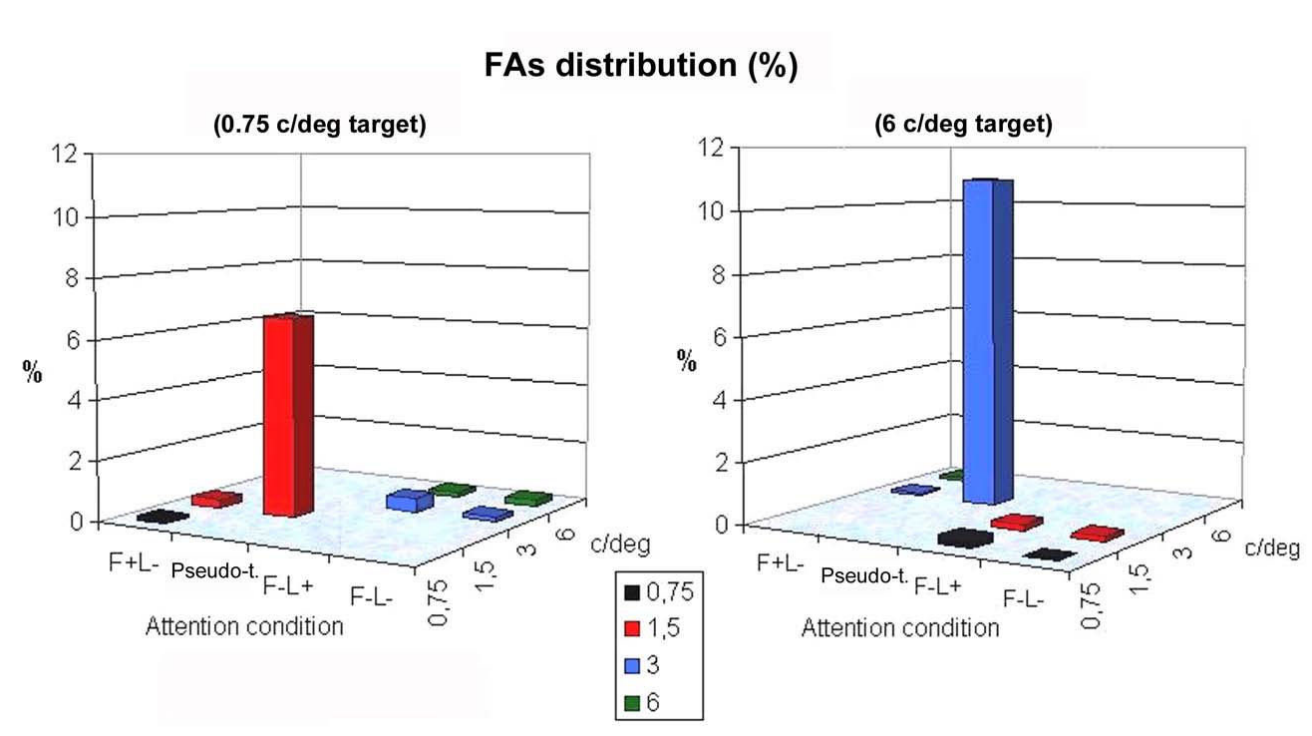
**False alarm distribution (percentages %) as a function of attention condition and grating spatial frequency**. It is evident that gratings falling at the attended location and within the target's spatial frequency bandwidth received the most of the FAs and were therefore considered as pseudo-targets.

The FA rate was extremely low and ranged from 0.2% to all types of non-targets (including those of the target frequency: 0.75 and 6 c/deg) to an average of 8.77% for pseudo-targets falling at the attended location (L+) and within the target spatial frequency bandwidth (F+/-), as illustrated in Figure 1 (6.63% for the attend-0.75 condition, and 10.90% for the attend-6 condition). The fact that gratings falling at the attended location and within the target's spatial frequency bandwidth (pseudo-targets) elicited 40 times more FAs than other types of lures indicates how similar they were to targets and how difficult they were to suppress at both the cognitive and response preparation levels.

**Figure 2:**
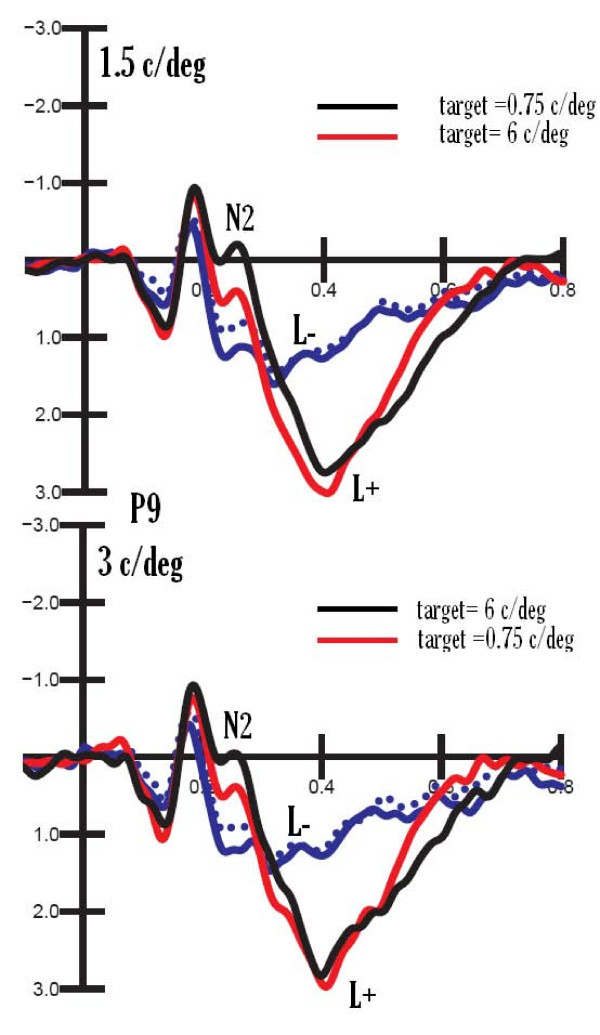
**Grand-average (N = 26) ERP waveforms recorded over the left occipito-temporal region in response to gratings of 1.5 and 3 c/deg, sharing or not sharing space location with the target (L+ or L-) and falling or not falling within the same spatial frequency bandwidth as the target**.

Figure 2 shows ERP waveforms recorded over posterior scalp sites in response to lure gratings of 1.5 and 3 c/deg, sharing or not sharing space location with the target (L+ or L-) and falling or not falling within the same spatial frequency bandwidth as the target. Strong frequency-based attentional effects are visible for both types of grating (especially at the *selection negativity *level), suggesting that when attention was paid to 0.75 c/deg the most similar gratings were 1.5 c/deg, and when attention was paid to 6 c/deg the most similar gratings were 3 c/deg in frequency.

**Figure 3:**
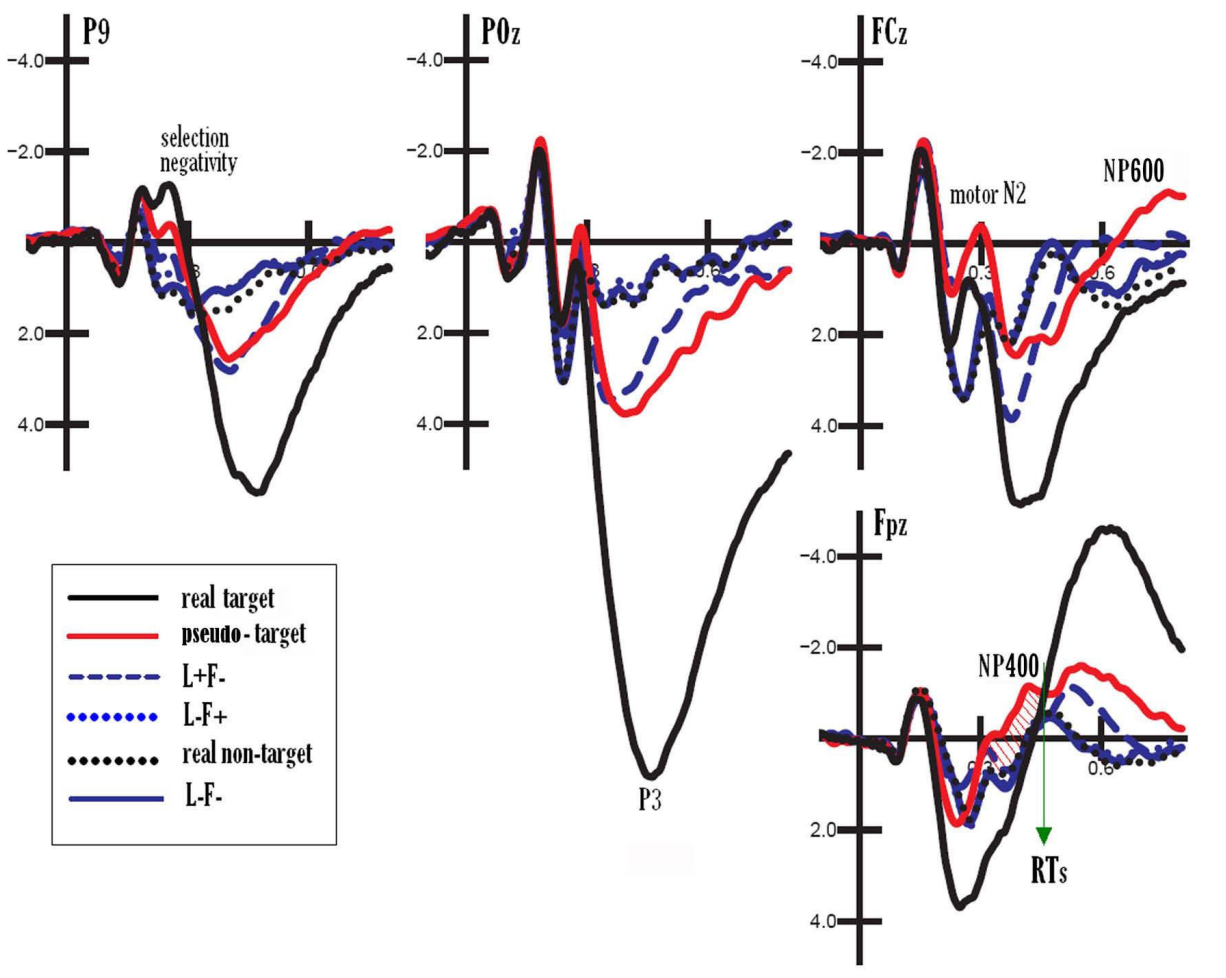
**Grand-average (N = 26) ERP waveforms recorded at left occipito/temporal (P9), midline occipital/parietal (POz), precentral (FCz) and prefrontal (PFz) sites as a function of attention condition and independent of stimulus spatial frequency**. ERPs elicited by real targets (the extreme frequencies 0.75 and 6 c/deg) are in black, whereas the ERPs elicited by lures are in colour. The only component elicited by pseudo-targets that did not clearly indicate targetness was the prefrontal N3 deflection.

In fact, ERP analysis revealed strong attentional effects for intermediate frequencies (lure stimuli) presented at the attended location and falling within the same spatial frequency bandwidth as actual targets (see Figure 3). They included typical attentional *selection negativity *over the occipito/temporal area (N2), a posterior P3 component, and a motor precentral N2 component larger to pseudo-targets than L+F- stimuli, and to the former categories than to location-irrelevant gratings. Even earlier C1 and P1 spatial frequency and location-relevant effects are visible from the grand-average ERPs, further supporting the evidence for early spatial frequency-based selective attention effects for target stimuli [20].

**Figure 4:**
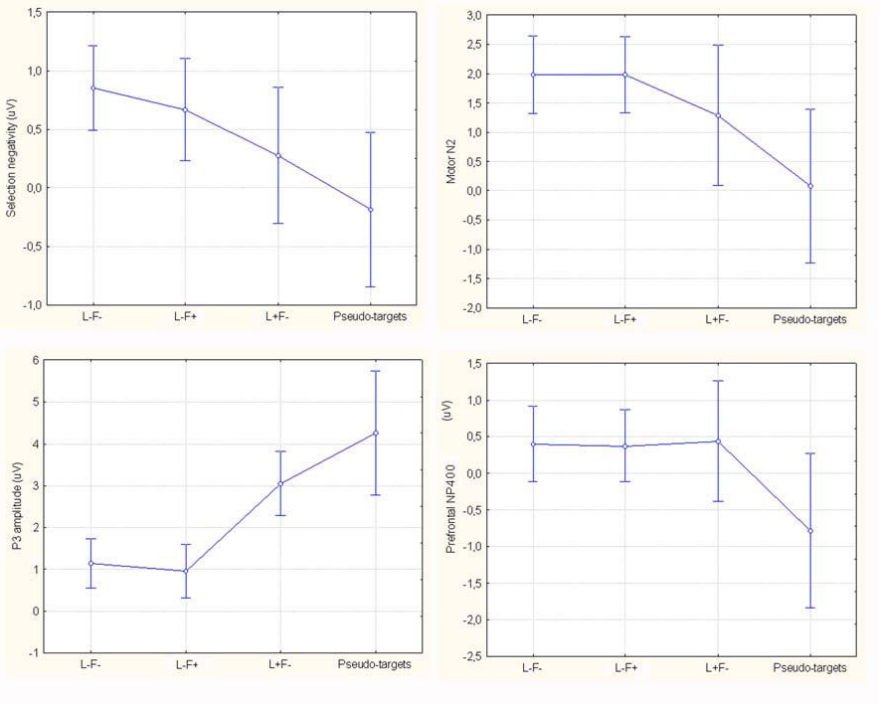
**Mean amplitude values (in μV) of ERP components of interest recorded as a function of attention condition and independent of grating spatial frequency**. Error bars reflect standard errors.

Statistical analyses performed on the mean area amplitude of posterior N2 (attentional *selection negativity*) showed that the attention condition was highly significant (F [3,75] = 16.06, GG adjusted p < 0.000059) with larger negativities to pseudo-targets than stimuli sharing only space location with the targets, and to the latter than to non-targets (see mean amplitude values in Figure 4). The attention condition was also strongly significant for the P3b posterior component (F [3,75] = 23.35, GG adjusted p < 0.00002), with larger positivities to pseudo-targets than stimuli sharing only space location with the targets, and to the latter than to other lures. P3b to pseudo-targets was of greater amplitude over the left than the right hemisphere, as indicated by the significant hemisphere × attention interaction and relative post-hoc comparisons (F [3,75] = 10.97 GG adjusted p < 0.000021). At precentral sites, the motor N2 potential was also affected by the attention condition (F [3,75] = 8.61, GG adjusted, p < 0.0026), with larger negativities to pseudo-targets than to all other distracters. Similarly, the prefrontal NP400 was strongly affected by the attention condition (F [3,75] = 6.48, GG adjusted, p < 0.00087), with larger negativities to pseudo-targets than to all other stimulus categories. Since mean reaction times occurred at about 500 ms of latency (mean RT = 510 ms) it was hypothesized that the NP400 prefrontal response (370–430 ms), the only potential markedly larger to the most-similar-as-possible-to-targets distracters (pseudo-targets), which were more difficult to suppress, might be considered a sign of neural suppression. Indeed, pseudo-targets showed all signs of being processed as targets except for this last sign of non-targetness (N3) followed by a lack of motor response.

**Figure 5:**
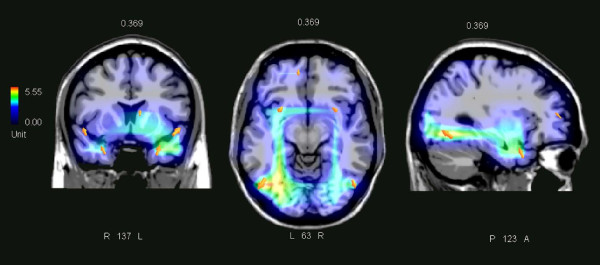
**swLORETA **[19]** inverse solution displaying the neural generators of the N3 suppression effect for pseudo-targets**. LORETA was computed on the difference wave obtained by subtracting ERPs to L-F+ from ERP to pseudo-targets in the time window 370–430 ms, corresponding to the maximum amplitude of the prefrontal N3 response. A realistic boundary element model (BEM) was derived from a T1 weighted 3D MRI data set by segmentation of the brain tissue. The BEM model consisted of one homogenic compartment made up of 3446 vertices and 6888 triangles. The head model was used for intra-cranial localization of surface potentials. Segmentation and head model generation were performed using the ASA (A.N.T. Software B.V., Enschede, the Netherlands) package [45]. The electromagnetic dipoles are shown as arrows and indicate the position, orientation and magnitude of dipole modelling solution applied to the ERP difference wave in the specific time window (370–430 ms). The different colours represent differences in the magnitude of the electromagnetic signal (in nA). 0.369 indicates the boundary of time window (370 ms at 512 Hz of sampling rate). L = left; R = right; P = posterior; A = anterior; numbers refer to the displayed brain slice in coronal, axial and sagittal views, respectively.

In order to investigate the neural bases of the suppression effect for pseudo-targets, a difference wave was computed by subtracting the ERPs to lures that were less difficult to suppress (frequency-pseudorelevant (F+) but location-irrelevant (L-)) from the ERPs to pseudo-targets (location-relevant and falling within the relevant spatial frequency bandwidth). A LORETA inverse solution was therefore performed on the difference wave in the time window 370–430 ms. The neural generators explaining the surface difference voltage are shown in Figure 5 and their Tailarach coordinates are listed in Table 1. The active sources included the left prefrontal cortex (BA9), the left and right superior temporal gyrus (BA38), and the left and right fusiform gyrus of the temporal cortex (BA19/20), with a left hemispheric asymmetry in the magnitude of activation (nA).

**Table 1:** Tailarach coordinates corresponding to the intracranial generators explaining the difference voltages related to the pseudo-target suppression effect in the 370–430 ms time window, according to swLORETA [18]; grid spacing = 10 mm, estimated SNR = 3.

**Magnit.**	**T-x [mm]**	**T-y [mm]**	**T-z [mm]**	**H**	**Lobe**	**Area**	**BA**
2.492	50.8	23.6	-22.9	RH	Temporal	Fusiform gyrus	20

3.726	31.0	8.2	-20.0	RH	Temporal	Superior Temp. gyrus	38

4.402	28.5	8.2	-20.0	LH	Temporal	Superior Temp. gyrus	38

4.165	50.8	-66.1	-10.5	RH	Temporal	Fusiform gyrus	19

5.549	-48.5	-66.1	-10.9	LH	Temporal	Fusiform gyrus	19

3.035	-8.5	44.4	15	LH	Frontal	Medial frontal gyrus	9

## Discussion

The aim of the study was to investigate the neural bases of executive control mechanisms involved in the ability to suppress irrelevant visual information and inappropriate motor responses. ERPs to intermediate irrelevant spatial frequencies were examined in the context of a conjoined space- and frequency-based selective attention task. The analysis of false alarm rates proved that non-target stimuli falling at the attended location (L+) and within the target spatial frequency bandwidth (pseudo-targets) were more difficult to suppress in that they elicited an average of 8.77% of FAs. Pseudo-target responses were characterized by a pronounced occipito/temporal *selection negativity *[16,21-23] indicating perceptual similarity to target gratings. At posterior sites, pseudo-targets elicited a large P3b component probably reflecting voluntary allocation of visual attention to targets as described as a key function of the parietal cortex by Cabeza and coworkers [24]. Consistent with this pattern of results, a marked N2 peak was visible at fronto-central sites, very probably indicating motor preparation processes [9,25]; it was of greater amplitude to pseudo-targets than to other distracters, which exhibited a sort of frontal P3 response instead. In this context, the frontal P3 cannot be interpreted as a sign of suppression, as in many go-no/go paradigms [1,10,26], since it was much lower in response to lures that were most difficult to suppress (i.e. pseudo-targets), as demonstrated by the false alarm distributions. Therefore, the present data do not support the view [2,8,9] that frontal P3 response to no-go trials might represent inhibition of responses with no-go stimuli. On the other hand, P3 might be conceptualized as a lack of motor preparation and of the negative voltage response execution processes typical of go trials [4,11], which were found in association with pseudo-target presentation, because of their striking similarity to real targets. In addition, it might also indicate a sort of P3b reflecting monitoring processing and stimulus evaluation. Furthermore, since no response was emitted to any of the lures considered in this study (since ERPs associated with incorrect trials were rejected), pseudo-targets and other distracters are perfectly comparable because they represent the brain processing of the same physical stimulus under different attention conditions, and the ERPs are not contaminated by overt motor responses as in go/no-go paradigms.

Inspection of the waveforms of Figure 3 reveals that motor N2 appeared larger to pseudo-targets than to targets, and one might be tempted to identify this component as the N2 response described in the oddball literature as being greater to no-go than go trials (e.g. [8]. However, this differential effect might very well be because the overlapping positivity (typical of space-relevant L+ stimuli) of pseudo-targets is lower than to real targets. Unfortunately, therefore, it cannot be demonstrated that frontal N2 to non-targets was a sign of motor suppression in this specific case, although the possibility cannot be excluded a priori.

At prefrontal sites a negative deflection (NP400) was identified in the 370–430 ms time window, which was not characteristic of target stimuli (as proved by a direct comparison with the ERPs elicited by real targets at Fpz, in Figure 3) and was much smaller in response to lures falling at an unattended location or outside the target spatial frequency bandwidth. For this reason, we hypothesized that the NP400 deflection might reflect the brain activity linked to the suppression of irrelevant information and/or the inhibition of inappropriate responses. The present study does not address the question of whether NP400 might indicate a suppression of motor or cognitive information, or conflict monitoring vs. response inhibition, but focuses on the finding of a clear sign that irrelevant information is suppressed without the problems inherent to the go/no-go paradigm.

**Figure 6:**
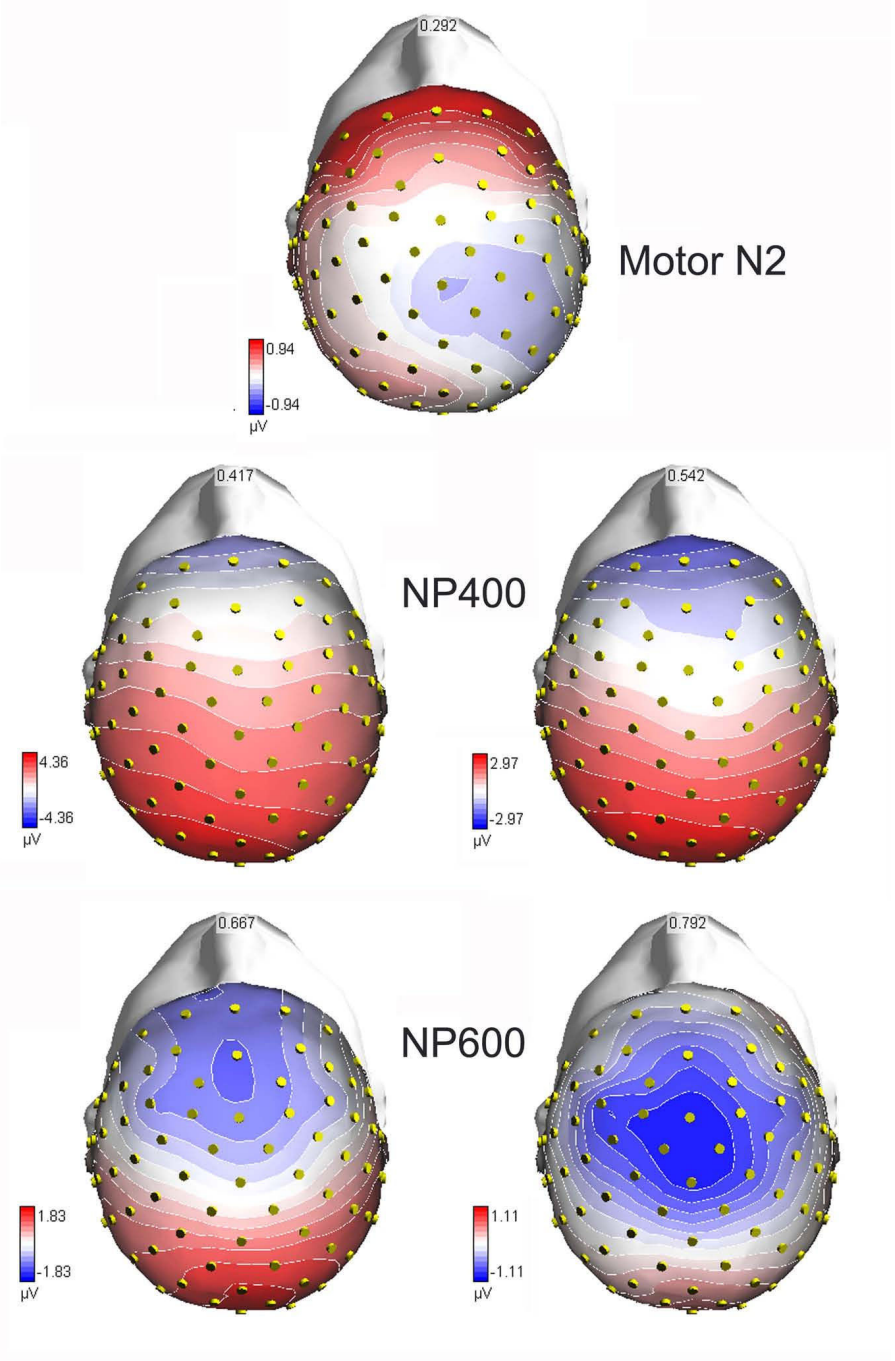
**Time series of voltage topographical maps (anterior-top view) relative to pseudo-target processing and computed every 125 from 292 ms to 792 ms**. Maps were made by plotting colour-coded isopotentials derived by interpolating voltage values between scalp electrodes at specific latencies.

In summary, notwithstanding the apparent similarity between motor N2 and NP400, the two components, one peaking at about 300 ms over precentral sites, the other at about 430 ms at prefrontal sites, were quite different in nature, the former (motor N2) being very pronounced in response to real targets and indicating targetness, and the latter being very pronounced in response to lures and indicating non-targetness). They also differed in terms of scalp distribution, as visible in topographical maps of Figure 6, displaying the voltage distribution of motor N2, prefrontal NP400 and frontal NP600 at anterior electrode sites.

**Figure 7:**
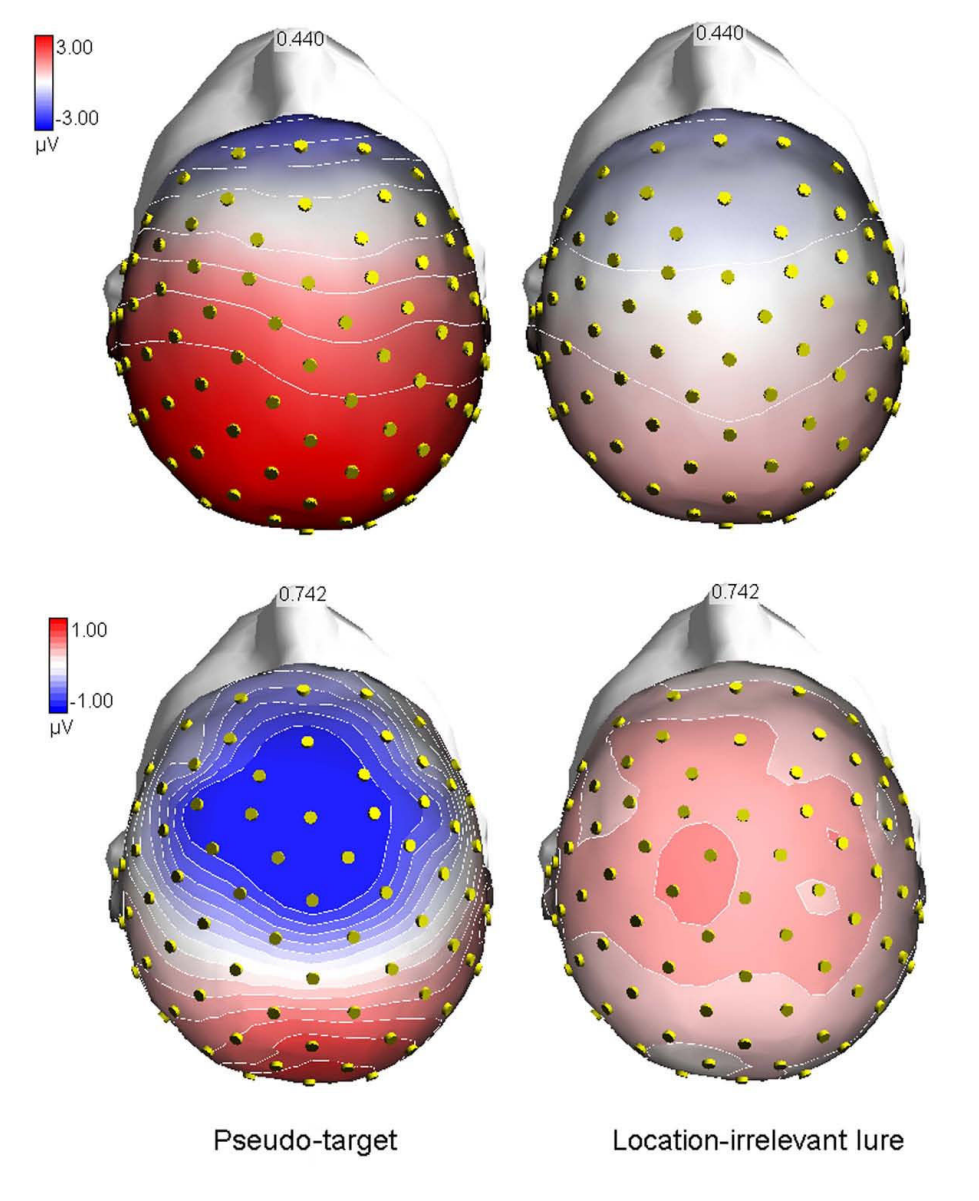
**Voltage topographical maps (anterior-top view) relative to pseudo-targets (left column) and L-F+ gratings (right column) processing**. As explained in the text, while prefrontal N400 very likely reflected cortical inhibition of lures difficult to suppress, the widespread positivity, which was clearly absent to non-targets, was probably a sign of (reduced) targetness of gratings falling in the same location and spatial frequency bandwidth as targets.

The enhanced NP400 to pseudo-targets than other lures less difficult to suppress persisted at frontal sites in the form of a large negative NP600 deflection. The evidence that this potential was still larger to pseudo-targets than other lures at both Fpz and FCz, and even larger to the former stimuli than real targets at FCz sites (see Figure 3) suggests a possible functional similarity with NP400, and its role in the sustained suppression of irrelevant visual information. The problem with NP600, however, is that it was larger in amplitude to targets than pseudo-targets at prefrontal sites, rendering it difficult to fully understand its functional meaning. Proponents of the no-go related frontal P3 might hypothesize that P400 (the wide-spread positivity visible in the left upper map of Figure 7), and not only prefrontal N400, is indeed a reflection of cortical inhibition of irrelevant stimuli. However, this hypothesis is countered by the evidence that P400 was much larger to target than pseudo-targets, therefore indexing stimulus selection rather than inhibition. Thus, the question remains unsolved.

It is interesting to note that the onset of prefrontal NP400, in our study, followed the stage of motor preparation indicated by precentral N2, and preceded the latency of response times (indicated by the green arrow in Figure 3) that corresponded to an average of 510 ms.

In another study, a negative event-related brain potential deflection (N470) was described, the generator of which was located in the anterior cingulate cortex; it was possibly related to response inhibition in a delayed response task [27] and was similar in morphology to the N430 described in the present conjoined selective visual attention task. However, while the anterior cingulate cortex seems most involved in the conflict monitoring [28,29] typical of go/no-go tasks, the prefrontal cortex seems more involved in the top-down modulation of attentional processes typical of conjoined selective attention tasks. Several neuroimaging and neurophysiological studies have provided evidence for a role of the dorsolateral prefrontal cortex (DLPF:BA9/46) in the suppression of motor behaviour [30-32] and in cognitive control [33,34].

Activation of the dorsolateral prefrontal cortex, during both inhibition of the prepotent impulse to respond and the suppression of irrelevant stimuli, is consistent with the view that this region is involved in cognitive control processes. Previous studies have reported a linear relationship between DLPFC activation and the degree of cognitive control [29,35]. Consistent with these data, Blasi and coauthors [31] found greater DLPFC activation during the relatively more difficult no/go condition (as reflected by performance scores that were poorer than under other conditions) than during the go condition.

It is also known that patients with prefrontal lesions confined to BA areas 9 and 46 are impaired in their ability not only to focus attention on task-relevant stimuli [36] but also to suppress task-irrelevant information [37]. In our study, the bilaterally increased occipito/temporal and superior temporal activation found in the LORETA inverse solution for the N3 suppression effect suggests a marked attentional modulation of the ventral stream, probably reflecting the activity of the ipsilateral fronto-temporal circuit described by Knight and other authors [38-40]. Indeed, it has been shown that the prefrontal cortex exerts modality-specific GABA-mediated suppression of sensory transmission through thalamic relay nuclei. The prefrontal cortex also has a role in response inhibition (the cognitive process required to cancel an intended movement), that is, in the suppression of inappropriate responses. In fact, it has been shown that the inferior frontal cortex is able to suppress an already-initiated manual response through a direct fronto-striatal pathway involving the subthalamic nucleus of the basal ganglia [41].

Our study revealed indications of hemispheric asymmetry in the modulatory attention effects: a greater posterior P3b component was found to pseudo-targets over the left than the right hemisphere. Again, swLORETA showed a left-sided activation of the left dorsolateral prefrontal cortex and an enhanced activation for pseudo-targets that was greater in the left than the right fusiform gyrus. Overall, these findings might suggest that the left hemisphere has better selective capability when dealing with gratings of similar spatial frequencies, so it requires a narrower attentional focus [42-44].

## Limitations

A possible limitation of the study, as suggested by one of our referees, is that the electrode material (pure tin) that is commonly used for EEG caps (Electro-Cap International, Inc.) might have induced a polarization for which late NP600 and, possibly, NP400 might represent the overshooting high-pass filtered P3b due to high impedance for slow oscillations. However we regard this suspicion as highly hypothetical.

## Conclusion

In summary, the combined observation of false alarms (FAs) and event-related potentials to distracter stimuli in a selective attention task to a conjunction of space location and spatial frequency of gratings showed that irrelevant stimuli that are more difficult to suppress (pseudo-targets) featured the ERP components typical of targets: occipito/temporal *selection negativity*, posterior P3b and precentral motor N2. In addition, they exhibited a large negativity at the prefrontal area (370–430 ms), following the motor preparation stage (275–315 ms) and preceding the reaction time stage (about 510 ms), which was the best candidate for reflecting response inhibition and top-down cognitive control. The swLORETA inverse solution [19,45] identified the neural generators of this effect in the left dorsolateral prefrontal cortex (BA9), left and right fusiform gyri (with left hemispheric asymmetry) and bilateral superior temporal cortices. We advance the hypothesis that these activations might reflect the modulatory effects exerted by the fronto/temporal circuit for the suppression of irrelevant information. However, further investigation will be certainly needed to corroborate any interpretation.

## Competing interests

The authors declare that they have no competing interests.

## Authors' contributions

AMP conceived of and coordinated the study, interpreted the data and drafted the manuscript. MDZ acquired, processed, and analyzed the ERP data, NC analyzed behavioural data, AZ was involved in the design of the paradigm, interpretation of data, and revision of the manuscript. All authors read and approved the final manuscript.
